# Epidemiology of *Salmonella* *enterica* Serotype Dublin Infections among Humans, United States, 1968–2013 

**DOI:** 10.3201/eid2309.170136

**Published:** 2017-09

**Authors:** R. Reid Harvey, Cindy R. Friedman, Stacy M. Crim, Michael Judd, Kelly A. Barrett, Beth Tolar, Jason P. Folster, Patricia M. Griffin, Allison C. Brown

**Affiliations:** Centers for Disease Control and Prevention, Atlanta, Georgia, USA

**Keywords:** Salmonella Dublin, antimicrobial resistance, zoonoses, bloodstream infections, bacteria, United States, Salmonella enterica, serotype Dublin

## Abstract

*Salmonella enterica* serotype Dublin is a cattle-adapted bacterium that typically causes bloodstream infections in humans. To summarize demographic, clinical, and antimicrobial drug resistance characteristics of human infections with this organism in the United States, we analyzed data for 1968–2013 from 5 US surveillance systems. During this period, the incidence rate for infection with *Salmonella* Dublin increased more than that for infection with other *Salmonella*. Data from 1 system (FoodNet) showed that a higher percentage of persons with *Salmonella* Dublin infection were hospitalized and died during 2005−2013 (78% hospitalized, 4.2% died) than during 1996–2004 (68% hospitalized, 2.7% died). Susceptibility data showed that a higher percentage of isolates were resistant to >7 classes of antimicrobial drugs during 2005–2013 (50.8%) than during 1996–2004 (2.4%).

## Abstract 2

Infection incidence and antimicrobial drug resistance are increasing.

*Salmonella* Dublin is a zoonotic *Salmonella* *enterica* serotype that in recent years has increased in infection incidence, antimicrobial drug resistance, and illness clinical severity. The Centers for Disease Control and Prevention (CDC) estimates that each year in the United States, *Salmonella enterica* causes 1.2 million infections, 24,000 hospitalizations, and 450 deaths (*1*). Although >2,500 serotypes of *Salmonella* exist (*2*), only ≈50 serotypes are regularly isolated from humans. Illnesses caused by nontyphoidal *Salmonella* are often self-limiting and require no antimicrobial drug therapy, but for patients with invasive infections, treatment is critical. Antimicrobial drug–resistant strains of *Salmonella* are associated with more severe illness and are more likely to result in bloodstream infection, hospitalization, and death than are illnesses caused by drug-susceptible strains (*3*,*4*). According to surveillance data from the National Antimicrobial Resistance Monitoring System (NARMS), the proportion of resistant isolates is higher among *S.* *enterica* serotype Dublin than among other serotypes (*5*).

Unlike most nontyphoidal *Salmonella* serotypes, which affect a broad spectrum of unrelated host species, *Salmonella* Dublin is a cattle-adapted serotype (*6*). The most comprehensive analysis of cases of *Salmonella* Dublin infection was published in 1982 and demonstrated that this serotype causes rare but severe disease in humans (i.e., bloodstream infection) that often requires antimicrobial drug therapy (*7*). Using available data across various CDC surveillance systems, we analyzed the epidemiology of human infections with *Salmonella* Dublin in the United States, including antimicrobial drug resistance, and compared it with that of other *Salmonella* serotypes.

## Methods

### Data Sources

#### Laboratory-based Enteric Disease Surveillance, 1968–2013

Begun in 1968, the CDC Laboratory-based Enteric Disease Surveillance system (LEDS) collects serotype and demographic data for every *Salmonella* isolate obtained from a human and submitted to US state and territorial public health laboratories. We used LEDS data to estimate national incidence rates (no. cases/100,000 population, using US census population estimates) of reported *Salmonella* Dublin and other nontyphoidal *Salmonella* serotypes. We excluded all typhoidal serotypes: Typhi, Paratyphi A, Paratyphi B (L[+] tartrate-negative), and Paratyphi C. We defined other nontyphoidal *Salmonella* as serotypes other than *Salmonella* Dublin (hereafter called other *Salmonella*). We also used LEDS data to evaluate differences in proportions of patients by race, ethnicity, and home state, infected with *Salmonella* Dublin and other *Salmonella* serotypes.

#### Foodborne Diseases Active Surveillance Network, 1996–2013

Since 1996, the Foodborne Diseases Active Surveillance Network (FoodNet) has conducted active, population-based surveillance for culture-confirmed cases of infection caused by 9 pathogens, including *Salmonella*, transmitted commonly through food in the United States. FoodNet is a collaboration of CDC, 10 state health departments, the US Department of Agriculture Food Safety and Inspection Service (USDA FSIS), and the US Food and Drug Administration (FDA). The FoodNet surveillance area includes 15% of the US population. For each reported case, FoodNet sites collect data on demographic characteristics, hospitalization, and outcome. Since 2004, FoodNet has also collected data on international travel (defined as travel abroad in the 7 days before illness began) and whether the case was associated with an outbreak. We used FoodNet data to compare demographics, clinical outcomes, and travel history among patients infected with *Salmonella* Dublin and those infected with other *Salmonella* serotypes.

#### National Molecular Subtyping Network for Foodborne Disease Surveillance, 1996–2013

Begun in 1996, the National Molecular Subtyping Network for Foodborne Disease Surveillance (PulseNet) is a national network of state and local public health laboratories and food regulatory agencies in the United States. Laboratorians upload pulsed-field gel electrophoresis patterns to PulseNet national databases. Comparison of these patterns enables identification of matches and possible outbreaks. The PulseNet database contains isolate data from human, food, environmental, and animal sources. We used PulseNet data to identify common nonhuman sources of *Salmonella* Dublin isolates.

#### Foodborne Disease Outbreak Surveillance System, 1973–2013

Since 1973, the Foodborne Disease Outbreak Surveillance System (FDOSS) has collected reports of enteric disease outbreaks transmitted by food in the United States. State and local public health agencies submit to CDC reports that include information about outbreak characteristics, food vehicles, and pathogens that caused each outbreak. We searched FDOSS data to describe the vehicles implicated in outbreaks of *Salmonella* Dublin infections.

#### NARMS, 1996–2013

Begun in 1996, NARMS is a collaboration among CDC, FDA, USDA, and state and local health departments. CDC asks public health laboratories that participate in LEDS to submit every 20th *Salmonella* isolate received from clinical laboratories to NARMS for the purpose of tracking changes in the antimicrobial susceptibility of certain enteric bacteria isolated from ill persons, retail meats, and food animals. We included NARMS data to compare antimicrobial resistance profiles (resistance to clinically important agents and the number of resistant classes) of *Salmonella* Dublin isolates from humans with those from other *Salmonella* serotypes. Susceptibility testing was conducted as previously described (*8*). In brief, isolates were tested for antimicrobial susceptibility by using broth microdilution (Sensititer; Trek Diagnostics, Cleveland, OH, USA) to determine the MIC for 14 antimicrobial agents (amikacin, gentamicin, streptomycin, ampicillin, amoxicillin/clavulanic acid, ceftiofur, ceftriaxone, cefoxitin, sulfamethoxazole/sulfisoxazole, trimethoprim/sulfamethoxazole, chloramphenicol, ciprofloxacin, nalidixic acid, and tetracycline). These agents were categorized into 8 classes, as defined by Clinical and Laboratory Standards Institute guidelines. When available, Clinical and Laboratory Standards Institute interpretive criteria were used to define resistance (*5*). A subset of isolates that showed resistance to ceftiofur or ceftriaxone were also tested for ceftazidime susceptibility. A multidrug-resistant (MDR) isolate was defined as one resistant to >3 classes of drug. We also examined specific resistance patterns, which included isolates that were resistant to at least ampicillin, chloramphenicol, streptomycin, sulfonamide (sulfamethoxazole/sulfisoxazole), and tetracycline (ACSSuT) and isolates that were also resistant to amoxicillin/clavulanic acid and ceftriaxone (ACSSuTAuCx). We compared antimicrobial resistance patterns of *Salmonella* Dublin between the 2 periods 1996–2004 and 2005–2013.

### Statistical Analyses

We used the Pearson χ^2^ test for statistical comparisons. We considered differences significant if the p value was <0.05. Statistical analyses were conducted by using SAS version 9.3 (SAS Institute, Cary, NC, USA).

## Results

### Incidence

**Figure 1:**
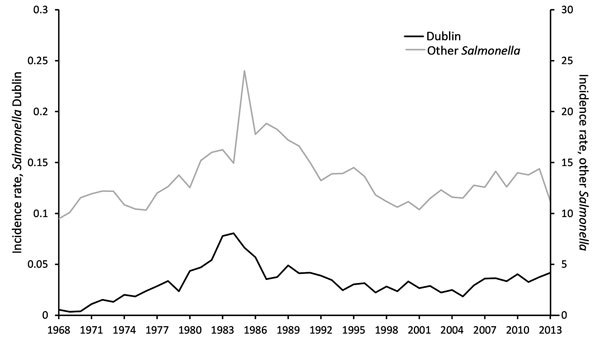
Incidence rates (no. cases/100,000 persons) for human infection with *Salmonella* *enterica* serotype Dublin and other nontyphoidal *Salmonella,* United States, 1968–2013. Data from the Centers for Disease Control and Prevention Laboratory-based Enteric Disease Surveillance system.

**Figure 2:**
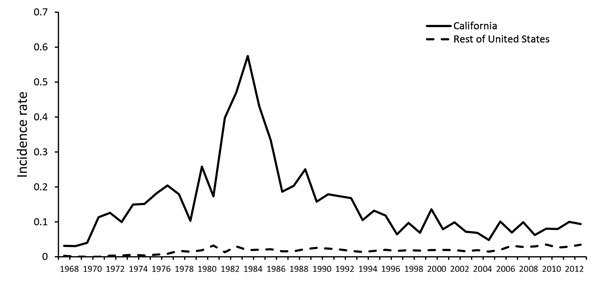
Incidence rates (no. cases/100,000 persons) for *Salmonella* *enterica* serotype Dublin infection in California and the rest of the United States, 1968–2013. Data from the Centers for Disease Control and Prevention Laboratory-based Enteric Disease Surveillance system.

During 1968–2013, states reported 3,903 cases of *Salmonella* Dublin infections to LEDS. These cases accounted for <0.25% of *Salmonella* infections reported. The incidence rate (no. *Salmonella* Dublin infections/100,000 persons) has been steadily rising since 1968 (0.0055 infections) with the exception of a distinct increase and subsequent decrease in incidence occurring throughout the 1980s, peaking in 1985 at 0.081 infections (Figure 1). The incidence rate for *Salmonella* Dublin infection was 7.6 times higher in 2013 (0.042 infections) than in 1968. In contrast, the incidence rate of other *Salmonella* infections has remained relatively stable since 1968 (9.5 infections compared with 11.2 infections in 2013). More than half (51%; 1,989/3,903) of all *Salmonella* Dublin infections were among California residents, including 74% (484/656) of infections during the peak in incidence from 1982 to 1985 (Figure 2). According to LEDS data, most *Salmonella* Dublin infections are reported from California; during 2005–2013, the 271 *Salmonella* Dublin infections reported from California accounted for 29% of the 943 cases reported to LEDS.

### Demographics

Demographics differed markedly among those infected with *Salmonella* Dublin and those with other *Salmonella*. According to FoodNet data, 38% of *Salmonella* Dublin infections occurred in persons >65 years of age, compared with 11% of other *Salmonella* infections (p<0.01) (Table 1). The median age of *Salmonella* Dublin patients was 55 years; the median age of patients with other *Salmonella* infections was 23 years (p<0.01). A total of 7% of *Salmonella* Dublin infections and 28% of other *Salmonella* infections occurred in children <5 years of age (p<0.01); 60% of *Salmonella* Dublin and 48% of other *Salmonella* infections occurred in men (p<0.01). We found no significant difference in history of international travel between patients with *Salmonella* Dublin (5%; 6/101) and other *Salmonella* infections (9%; 4,297/46,764) (p = 0.15).

**Table 1:** Demographics, international travel, clinical outcomes, and isolate source for *Salmonella enterica* serotype Dublin and other *Salmonella*, United States, 1996–2013*

Patient characteristics	*Salmonella* Dublin, no. (%), n = 228	Other *Salmonella*, no. (%), n = 97,814	p value
Demographics			
Age group, y†			
<1	2/228 (0.9)	11,075/97,562 (11.4)	<0.01
1–4	13/228 (5.7)	16,481/97,562 (16.9)	<0.01
5–17	8/228 (3.5)	15,628/97,562 (16.0)	<0.01
18–64	119/228 (52.2)	43,819/97,562 (44.9)	<0.05
>65	86/228 (37.7)	10,559/97,562 (10.8)	<0.01
Sex			
M	137/228 (60.1)	46,909/97,486 (48.1)	<0.01
F			
International travel	6/101 (5.0)	4,297/46,764 (8.6)	0.15
Clinical outcome			
Died	8/216 (3.7)	431/86,977 (0.5)	<0.01
Hospitalized‡	167/223 (74.9)	24,187/88,748 (37.3)	<0.01
Isolate source			
Blood	137/226 (60.7)	5,054/97,142 (5.2)	<0.01
Feces	49/226 (21.7)	8,6257/97,142 (88.8)	<0.01
Other	40/226 (17.7)	5,831/97,142 (6.0)	<0.01

*Data from the Foodborne Diseases Active Surveillance Network. †Median ages: *Salmonella* Dublin 55 (range <1–97) y; other *Salmonella* 23 (range <1–110) y; p<0.01. ‡Median hospital stays: *Salmonella* Dublin, 6 d (range 1‒76 d); other *Salmonella*, 3 d (range 0‒374 d); p<0.01.

### Clinical Outcomes and Severity of Disease

According to FoodNet data, *Salmonella* Dublin was more commonly isolated from blood (61%) than were other *Salmonella* (5%) (p<0.01) (Table 1). Hospitalization was reported for 75% of patients with *Salmonella* Dublin infection and 27% of patients with other *Salmonella* infections (p<0.01) (Table 1). Hospitalization lasted a median of 6 days for patients with *Salmonella* Dublin infection and 3 days for patients with other *Salmonella* infections (p<0.01). *Salmonella* infection resulted in death for 4% of patients with *Salmonella* Dublin infection and 0.5% of patients with other *Salmonella* infections (p<0.01).

**Figure 3:**
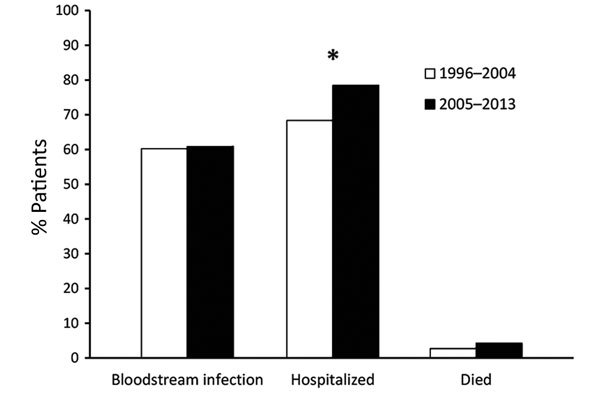
Percentage of patients with adverse clinical outcomes after infection with *Salmonella* *enterica* serotype Dublin, United States, 1996–2004 and 2005–2013. *p<0.05 (significant difference).

The proportion of *Salmonella* Dublin isolates from blood remained relatively constant during 1996–2004 (60%) and 2005–2013 (61%) (Figure 3). Hospitalization among *Salmonella* Dublin patients increased from 68% during 1996–2004 to 78% during 2005–2013 (p<0.05). The mortality rate increased from 2.7% during 1996–2004 to 4.2% during 2005–2013 (p = 0.57).

### Sources

#### Food and Animals

According to the PulseNet database, 478 *Salmonella* Dublin isolates were obtained from food during 1999–2013. Source data for 475 foodborne isolates indicated that 473 (99%) were from beef, 1 was from cooked pork, and 1 was from chili pepper. During this same period, another 376 *Salmonella* Dublin isolates were obtained from animals. Of the 331 of these isolates with source data available, 328 (99%) were from cattle and 3 were from a pig, a dog, and a horse.

#### Outbreaks

During 1973–2013, a total of 9 *Salmonella* Dublin outbreaks were reported to FDOSS. These outbreaks occurred in California (5 outbreaks), Washington (2), Arkansas (1), and Wisconsin (1). Of the 9 outbreaks, 6 (67%) occurred before 1982. For each of 3 outbreaks, the foodborne vehicle was identified (raw beef, raw milk, and Mexican-style cheese).

#### Sporadic Illnesses

We used LEDS data to determine the proportion of all *Salmonella* infections reported during 2007–2012. *Salmonella* Dublin infection was more common in states where the sale of raw milk is legal (328 cases/100,000 persons) (*9*) than in states where such sale is illegal (108 cases/100,000 persons) (p<0.01).

### Antimicrobial Resistance

During 1996–2013, a total of 102 clinical isolates of *Salmonella* Dublin were tested by NARMS (Table 2). Of these 102 isolates, 42 (41%) were pansusceptible; of the 33,415 isolates from other *Salmonella*, 26,552 (79%) were pansusceptible (p<0.01). Ceftriaxone resistance increased from detection in 0 of 5 isolates in 1996 to detection in 11 (92%) of 12 isolates in 2013 and was higher among *Salmonella* Dublin isolates (31%; 32/102) than among other *Salmonella* isolates (3%; 947/33,415) (p<0.01). Of the 31 ceftriaxone-resistant isolates that were also tested for ceftazidime resistance, 28 (90%) were resistant.

**Table 2:** Antimicrobial drug resistance in *Salmonella* *enterica* serotype Dublin and other *Salmonella*, United States, 1996–2013*

Resistance pattern	*Salmonella* Dublin, no. (%), n = 102	Other *Salmonella*, no. (%), n = 33,415
Pansusceptible	42 (41)	26,552 (79)
Resistant to >1 class	60 (59)	6,863 (21)
Resistant to >3 classes	56 (55)	4,013 (12)
Resistant to >5 classes	47 (46)	2,374 (7)
Resistant to >7 classes	32 (31)	601 (2)
Resistant to at least ACSSuT†	42 (41)	2,156 (6)
Resistant to at least ACSSuTAuCx‡	29 (28)	581 (2)
Resistant to ceftriaxone	32 (31)	947 (3)
Resistant to nalidixic acid	6 (6)	643 (2)
Resistant to nalidixic acid and ceftriaxone	4 (4)	39 (0.1)

*Data from the National Antimicrobial Resistance Monitoring System. p<0.01 for all. †Resistant to ampicillin, chloramphenicol, streptomycin, sulfamethoxazole/sulfisoxazole,and tetracycline. ‡Resistant to ACSSuT, amoxicillin–clavulanic acid, and ceftriaxone.

Multidrug resistance was found for 56 (55%) of *Salmonella* Dublin isolates compared with 4,013 (12%) of other *Salmonella* isolates (p<0.01) (Table 2). Among MDR *Salmonella* Dublin isolates, 84% were resistant to >5 classes of antimicrobial drugs and 57% were resistant to >7 classes; among MDR isolates of other *Salmonella*, 59% (p<0.01) were resistant to >5 classes and 15% (p<0.01) were resistant to >7 classes. ACSSuT resistance was found in 41% of *Salmonella* Dublin isolates, compared with 7% of other *Salmonella* isolates (p<0.01).

**Figure 4:**
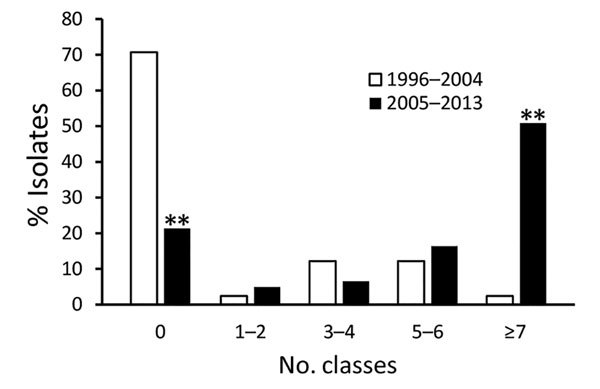
Number of Clinical Laboratory Standards Institute classes of antimicrobial drugs to which *Salmonella* *enterica* serotype Dublin isolates were resistant, 1996–2004 (n = 41) and 2005–2013 (n = 61). **p<0.01 (significant difference).

ACSSuTAuCx resistance was found for 28% of *Salmonella* Dublin isolates and 2% of other *Salmonella* isolates (p<0.01). Resistance to nalidixic acid was found for 6% of *Salmonella* Dublin isolates and 2% of other *Salmonella* isolates (p<0.01). Among nalidixic acid–resistant isolates, 67% of *Salmonella* Dublin isolates and 6% of other *Salmonella* isolates were also resistant to ceftriaxone (p<0.01). The proportion of *Salmonella* Dublin isolates resistant to antimicrobial drugs increased markedly from 1996–2004 to 2005–2013, from 29% to 79% for resistance to >1 classes (p<0.01) and from 2% to 51% for resistance to >7 antimicrobial classes (p<0.01) (Figure 4). Among resistant isolates, the median number of classes to which isolates were resistant increased from 4.5 to 7.0 (p<0.01). Resistance to ceftriaxone increased from 3% during 1996–2004 to 52% during 2005–2013 (p<0.01), and resistance to nalidixic acid increased from 0 to 10% during these same periods (p<0.05).

## Discussion

Decades of CDC surveillance data analyzed in this study illustrate that *Salmonella* Dublin more often causes bloodstream infections, hospitalizations (with longer hospital stays), and deaths than other *Salmonella* serotypes. Our findings support previous descriptions of *Salmonella* Dublin as a cattle-adapted serotype (*10*).

In the past decade, more than half of *Salmonella* Dublin infections have been resistant to >7 antimicrobial drug classes, and clinical outcomes have been more severe. The proportion of *Salmonella* Dublin isolates that were resistant was ≈2.7 times greater during 2005–2013 than during 1996–2004; isolates from the later period were also resistant to more antimicrobial drug classes. Multidrug resistance probably has direct clinical implications because bloodstream infections that require antimicrobial therapy tend to develop in patients with *Salmonella* Dublin infections (*3*). Most *Salmonella* Dublin isolates were resistant to third-generation cephalosporins (including ceftriaxone), which are often the treatment of choice for children with bloodstream infections because of the contraindication for fluoroquinolone use in children.

Clinical severity of *Salmonella* Dublin infections, as measured by the proportion of hospitalizations and deaths, also increased between 1996‒2004 and 2005‒2013. Our study did not directly measure the association between antimicrobial resistance and clinical severity of *Salmonella* Dublin infection by linking isolate data to outcome data. Nevertheless, by comparing both measures over the 2 periods, we showed that, for *Salmonella* Dublin infections, antimicrobial drug resistance and clinical severity increased in parallel. In addition to the older age of patients and concurrent conditions often associated with *Salmonella* Dublin infections (*7*,*11*), we hypothesize that the multidrug resistance profile has led to the higher rates of treatment failure, prolonged hospitalizations, and higher mortality rates observed in our study.

Virulence factors may also contribute, particularly those factors located on resistance plasmids that are co-selected for when antimicrobial drugs are used in cattle. *Salmonella* Dublin has been described as having a serotype-specific virulence-associated plasmid that is associated with invasive infection and remains stable through multiple generations of nonselective bacterial passage (*12*). Additional analyses, with use of whole-genome sequencing, particularly methods like those developed by Pacific Biosciences (Menlo Park, CA, USA) to use long-sequence reads and facilitate plasmid analysis, would enable investigation into the respective contributions of virulence factors and resistance mechanisms.

The recently observed increase in human infections with *Salmonella* Dublin resistant to ceftriaxone and nalidixic acid probably resulted, in part, from the agricultural use of comparable antimicrobial drugs in animals. Over the past 15 years, ceftriaxone resistance among *Salmonella* Dublin isolates from FSIS-PR/HACCP (Pathogen Reduction/Hazard Analysis and Critical Control Point) samples from cattle increased from 0 to 86% (*13*). Davis et al. determined that among *Salmonella* Dublin isolates from cattle, resistance to the third-generation cephalosporin ceftazidime increased over a 5-year period; they suggested that antimicrobial resistance in *Salmonella* Dublin is probably driven by antimicrobial drug use in cattle without influence of antimicrobial drug use in humans (*14*). Berge et al. also observed increasing resistance to third-generation cephalosporins and fluoroquinolones in calves in California during 1998–2002 (*15*). These findings demonstrate that antimicrobial stewardship and judicious use programs are essential for maintaining the efficacy of drugs used in human and veterinary medicine.

Our data indicate that the incidence of *Salmonella* Dublin infections has increased while the incidence of other *Salmonella* infections has remained mostly stable (Figure 1). The peak in overall *Salmonella* infections that occurred in the mid-1980s was driven by a nationwide outbreak of *Salmonella* Typhimurium (*16*). The simultaneous spike in *Salmonella* Dublin resulted largely from consumption of raw milk (*7*,*17*), particularly from a large California dairy (*18*). The dairy producer promoted its raw milk as having health benefits (*19*), and many persons with compromised immune systems (e.g., young, elderly, or HIV-positive) became ill (*20*). As a result of a public health investigation (*17*), production and sales were halted, and FDA banned the interstate sale of raw milk in 1987 (*21*). A sharp decline in *Salmonella* Dublin infections soon followed.

Although additional data on food histories and the role of the environment will help elucidate the sources of human infections, the risk for *Salmonella* Dublin infection among humans is probably still caused, in part, by consumption of raw milk and beef. Raw dairy products have been linked to numerous *Salmonella* Dublin illnesses in the United States (*17*,*22*) and abroad (*23*,*24*). Incidence of *Salmonella* Dublin infections was 3 times higher in states that allow the sale of raw milk or permit cow shares than in states where raw milk sales are illegal. US surveillance data from PulseNet, the USDA Agricultural Marketing Service (*25*), and FDOSS also indicate that *Salmonella* Dublin has been isolated from ground beef and boneless beef products and has been associated with outbreak-associated illnesses from beef products. *Salmonella* Dublin has also been found in beef cattle and calves (*26*,*27*).

In our study, the higher proportion of *Salmonella* Dublin infections among men than women may be partially attributable to consumption patterns. Although the 2006–2007 FoodNet Population Survey found no differences by sex for consumption of raw milk or cheese items (*28*), numerous studies have found that men consume more beef and more undercooked beef than women (*28*,*29*). Occupational exposure to cattle may also contribute to the increased frequency of infection among men.

Most *Salmonella* Dublin infections continue to be reported from California (Figure 2), but illnesses have occurred nationwide. They are probably associated with an ongoing outbreak of *Salmonella* Dublin infections among US dairy and beef cattle. A 2014 dairy study conducted by the USDA National Animal Health Monitoring System found antibodies directed against *Salmonella* Dublin lipopolysaccharide O-antigens in 8% of bulk tank milk samples (*30*). Of operations in participating western states (California, Colorado, Idaho, Texas, and Washington), 52% were positive, compared with 2.8% of operations in eastern states (Kentucky, Michigan, Minnesota, Missouri, New York, Ohio, Pennsylvania, Vermont, Virginia, Wisconsin). In 2013, the Animal Health Diagnostic Center at Cornell University (Ithaca, NY, USA) issued an animal health advisory, warning cattle owners about an increase in MDR *Salmonella* Dublin infections among cattle in the northeastern United States, treatment difficulties associated with these infections, the potential for long-term environmental contamination, and the dangers (including death) that these infections pose to animals and humans (*31*).

Changes in the geographic distribution of *Salmonella* Dublin infections in cattle probably explain the similar geographic spread among humans. Historically, *Salmonella* Dublin in cattle was associated with the western United States and was not discovered in cattle east of the Rocky Mountains until 1968 (*32*). *Salmonella* Dublin continued to spread by transport of animals and their products and can now be found in cattle populations throughout the contiguous United States (*26*).

In Denmark, in response to the specific threat to human and animal health posed by *Salmonella* Dublin infections, in 2006, the Danish government passed legislation intended to eradicate this serotype. Their policy actions included heightened surveillance for cattle and abattoirs, voluntary interventions to reduce environmental contamination and disease spread within infected herds, economic sanctions for producers who do not control *Salmonella* Dublin in their herds, and closing of infected herds to live-animal trade (*33*,*34*). In the United States, precedent for the successful eradication of other host-adapted *Salmonella* serotypes in production animals has been set by use of vaccines and improved management practices. An example is the USDA National Poultry Improvement Plan, which has successfully eradicated *Salmonella* Gallinarum and Pullorum from domestic commercial poultry (*35*,*36*). Efforts are under way to decrease the burden of *Salmonella* Dublin among cattle. An oral modified-live *Salmonella* Dublin vaccine has been evaluated for use in calves; however, this vaccine has not been effective for reducing the incidence of disease, and research into finding an effective vaccine continues (*37*).

Interventions developed for the Denmark cattle and US poultry industries may not be completely applicable to the US cattle industry because of regulatory and production differences. For example, in Denmark, to control *Salmonella* Dublin infections, trade restrictions are applied to farms with affected herds, and in the United States, biosecurity procedures for poultry producers generally enable tighter environmental control than do those for cattle producers. However, judicious use of antimicrobial drugs in cattle, coupled with improved specific husbandry and management practices on the farm, could decrease antimicrobial-resistant *Salmonella* Dublin infection in cattle. In 2012, FDA prohibited certain extralabel uses of cephalosporins in chickens, turkeys, cattle, and swine (*38*). This new prohibition has the potential to slow the spread of cephalosporin resistance among food animals and is a valuable step toward protecting the effectiveness of current antimicrobial drugs. Nevertheless, other extralabel uses of cephalosporin drugs are still permitted.

*Salmonella* Dublin is a cattle-adapted *Salmonella* serotype that causes severe and antimicrobial drug–resistant infections in humans and cattle, and its incidence is on the rise. Reducing *Salmonella* Dublin carriage by cattle could benefit animal and human health. Unlike most other *Salmonella* infections in food animals, *Salmonella* Dublin can cause high mortality rates, particularly among calves, and heavy economic burdens for producers (*39*). It is well established that use of antimicrobial agents is a major driving force for the global surge in antimicrobial resistance. Food animal management practices, including veterinary use of antimicrobial drugs, may be contributing to the increasing resistance in *Salmonella* Dublin and to *Salmonella* Dublin–associated illness and death among humans (*15*). Therefore, careful evaluation of management practices and judicious use of antimicrobial drugs in cattle is critical for the control of antimicrobial drug–resistant *Salmonella* Dublin infections in cattle and humans. The 2016 FDA Veterinary Feed Directive aims to eliminate the use for food production purposes (i.e., growth promotion and feed efficiency) of antimicrobial drugs that are considered medically important in humans and seeks to bring all remaining therapeutic use of antimicrobial agents in feed and water under the oversight of licensed veterinarians (*40*). Agricultural and public health authorities will need to engage in ongoing, meaningful collaborations to reduce inappropriate antimicrobial use in food-producing animals to protect human and animal health.
